# Increased Renal Methylglyoxal Formation with Down-Regulation of PGC-1α-FBPase Pathway in Cystathionine γ-Lyase Knockout Mice

**DOI:** 10.1371/journal.pone.0029592

**Published:** 2011-12-22

**Authors:** Ashley A. Untereiner, Arti Dhar, Jianghai Liu, Lingyun Wu

**Affiliations:** 1 Department of Pharmacology, College of Medicine, University of Saskatchewan, Saskatoon, Saskatchewan, Canada; 2 Department of Health Sciences, Lakehead University and Thunder Bay Regional Research Institute, Thunder Bay, Ontario, Canada; Virginia Commonwealth University Medical Center, United States of America

## Abstract

We have previously reported that hydrogen sulfide (H_2_S), a gasotransmitter and vasodilator has cytoprotective properties against methylglyoxal (MG), a reactive glucose metabolite associated with diabetes and hypertension. Recently, H_2_S was shown to up-regulate peroxisome proliferator-activated receptor-γ coactivator (PGC)-1α, a key gluconeogenic regulator that enhances the gene expression of the rate-limiting gluconeogenic enzyme, fructose-1,6-bisphosphatase (FBPase). Thus, we sought to determine whether MG levels and gluconeogenic enzymes are altered in kidneys of 6–22 week-old cystathionine γ-lyase knockout (CSE^-/-^; H_2_S-producing enzyme) male mice. MG levels were determined by HPLC. Plasma glucose levels were measured by an assay kit. Q-PCR was used to measure mRNA levels of PGC-1α and FBPase-1 and -2. Coupled-enzymatic assays were used to determine FBPase activity, or triosephosphate levels. Experimental controls were either age-matched wild type mice or untreated rat A-10 cells. Interestingly, we observed a significant decrease in plasma glucose levels along with a significant increase in plasma MG levels in all three age groups (6–8, 14–16, and 20–22 week-old) of the CSE^-/-^ mice. Indeed, renal MG and triosephosphates were increased, whereas renal FBPase activity, along with its mRNA levels, were decreased in the CSE^-/-^ mice. The decreased FBPase activity was accompanied by lower levels of its product, fructose-6-phosphate, and higher levels of its substrate, fructose-1,6-bisphosphate in renal extracts from the CSE^-/-^ mice. In agreement, PGC-1α mRNA levels were also significantly down-regulated in 6-22 week-old CSE^-/-^ mice. Furthermore, FBPase-1 and -2 mRNA levels were reduced in aorta tissues from CSE^-/-^ mice. Administration of NaHS, a H_2_S donor, increased the gene expression of PGC-1α and FBPase-1 and -2 in cultured rat A-10 cells. In conclusion, overproduction of MG in CSE^-/-^ mice is due to a H_2_S-mediated down-regulation of the PGC-1α-FBPase pathway, further suggesting the important role of H_2_S in the regulation of glucose metabolism and MG generation.

## Introduction

Hydrogen sulfide (H_2_S) is the most recent addition to the endogenous gasotransmitter family that includes nitric oxide (NO) and carbon monoxide (CO). H_2_S has remarkable vasodilatory [1], anti-inflammatory [2], and anti-oxidant properties [3]–[5]. This gasotransmitter is produced by cystathionine β-synthase, which is predominantly expressed in the brain and CNS, cystathionine γ-lyase (CSE), the predominant H_2_S-producing enzyme in the cardiovascular system [6], and by a newly identified enzyme, 3-mercaptopyruvate sulfurtransferase localized in the brain [7] and endothelium [8]. Recently, we showed that CSE deficiency and reduced endogenous H_2_S production in vascular tissues resulted in the development of hypertension in CSE^–/–^ mice [1].

Increased methylglyoxal (MG) has been linked to the development of insulin resistance, type 2 diabetes mellitus (T2DM) [9], [10], as well as hypertension [11]–[15]. As a member of the reactive carbonyl species, MG is formed mainly through the non-enzymatic conversion of triosephosphates, such as dihydroxyacetone phosphate (DHAP) and glyceraldehyde 3-phosphate (GA3P) [16], [17]. The triosephosphate pool, in turn, is regulated by cellular levels of glucose and fructose. Interestingly, recent studies showed that plasma fructose level, not glucose, is more involved in MG overproduction [14], [17], which is mainly attributed to the over-activated polyol pathway [17]. As such, the importance of glycolysis in MG overproduction during hyperglycaemia is questioned, because elevated levels of MG in the plasma, serum, and aorta was shown to occur under normoglycemic conditions in spontaneously hypertensive rats [13], [14] and in Zucker obese rats [17]. In our most recent study, we demonstrated that MG lowers H_2_S concentrations in cultured vascular smooth muscle cells (VSMCs) both directly by scavenging H_2_S and indirectly by down-regulating CSE expression [3], suggesting an important interaction between MG and H_2_S. As a reciprocal, it is likely that low H_2_S levels may result in elevated MG levels.

The kidney plays a vital role in blood pressure regulation [18], but its role in glucose metabolism is often ignored [19]. Indeed, renal gluconeogenesis has been estimated to account for 20±2% of total glucose release [19], where under diabetic conditions this is dramatically increased [19], [20]. The rate of gluconeogenesis is mainly regulated by the activities of certain unidirectional enzymes, notably phosphoenolpyruvate carboxykinase (PEPCK), fructose-1,6-bisphosphatase (FBPase), and glucose-6-phosphatase [21]. Peroxisome proliferator-activated receptor-γ coactivator (PGC)-1α is a key regulator of energy metabolism [22] and is a strong coactivator of PEPCK, FBPase, and the orphan nuclear receptor estrogen-related receptor-α (ERRα), which in turn mediates PGC-1α activity [23]. Interestingly, NO has been shown to increase PGC-1α expression in adipocytes and HeLa cells [24], and similar findings have been reported for CO in mouse hearts [25]. However, it has yet to be determined if H_2_S can also alter PGC-1α expression.

The present study investigated whether MG level was altered in CSE^–/–^ mice and its underlying mechanisms. To this end, we measured plasma and renal MG levels in both CSE^+/+^ and CSE^–/–^ mice at different age groups (6–22 weeks). We also evaluated the role of FBPase and related signaling pathway in the regulation of MG formation.

## Materials and Methods

### Animals and Tissue Preparation

Male 6–22 week-old CSE^+/+^ and CSE^–/–^ mice (C57BL/6J x 129SvEv) were in-house bred as we described previously [1]. These animals were housed in a temperature-regulated animal facility, exposed to a 12 h light/dark cycle with free access to food and water. The Animal Health Care Committee of the University of Saskatchewan specifically approved this animal study (protocol number: 20030085). Prior to harvesting tissues, mice were starved for 16 h. Kidneys and aortas were isolated in ice-cold PBS, cleaned, and snap-frozen in liquid nitrogen immediately. Tissues were pulverized with a Mikro-Dismembrator (B. Braun Biotech International, PA, USA) and stored at −80°C until processing.

### Cell culture

Rat aortic smooth muscle cell line (A-10 cells) was obtained from American Type Culture Collection and cultured in Dulbecco's modified Eagle's medium (DMEM) in a humidified atmosphere of 95% air and 5% CO_2_ as described [3]. Cultured cells were starved in serum-free DMEM for 24 h and exposed to NaHS treatment for 24 h. Cells were then washed with ice-cold PBS, harvested by trypsinization, and resuspended in cell lysis buffer supplied by RNeasy Mini Kit (Qiagen sciences, MD, USA).

### Plasma glucose measurement

Plasma glucose levels were determined by using the QuantiChrom™ Glucose Assay Kit (BioAssay Systems, USA), and followed accordingly to the manufacturer's instructions. Briefly, 5 µL of sample was mixed with 500 µL of Reagent, and then placed on a heating block set at 100°C for 8 min. After cooled to room temperature, samples were transferred to 96-well plate and the absorbance was read at 630 nm in a Multiskan Spectrum (Thermo Labsystems). Samples were measured in triplicate and calibrated by comparison with the given manufacturer standards.

### MG measurement

Quantitation of MG was performed by the widely accepted *o*-phenylenediamine (*o*-PD)-based assay as described previously [26]. Kidney samples were prepared in 50 mM sodium phosphate monobasic buffer (pH 4.5) and sonicated twice for 15 s on ice, then centrifuged at 12,000 rpm at 4°C for 10 min. A portion of the supernatant was used for protein determination *via* the bicinchoninic acid (BCA) procedure. The supernatant of kidney homogenate was incubated with a final concentration of 10 mM *o*-PD (derivatizing agent) and 0.45 N perchloric acid (PCA) with 50 µM EDTA for 24 h at room temperature and protected from light. The quinoxaline formed between dicarbonyl compounds and *o*-PD, as well as the internal standard (5-methylquinoxaline) were measured using a Hitachi D-7000 high performance liquid chromatography (HPLC) system (Hitachi Ltd., Ontario, Canada). A Nova-Pak C18 column was used (Waters, MA, USA). The mobile phase was composed of 8% (v/v) of 50 mM NaH_2_PO_4_ (pH 4.5), 17% (v/v) of HPLC grade acetonitrile and 75% of water. Samples were measured in triplicate and calibrated by comparison with a 2-methylquinoxaline standard.

### H_2_S measurement in plasma and kidney tissues

The measurement of renal H_2_S level followed the established protocol in our laboratory [1], [3], [27], [28]. Kidney homogenates were suspended in 50 mM ice-cold potassium phosphate buffer (pH 6.8) and added to a reaction mixture containing (mM): 100 potassium phosphate buffer (pH 7.4), 10 L-cysteine, and 2 pyridoxal-5′-phosphate. The reaction was performed in a 25 mL Erlenmeyer flask (Pyrex, USA). Cryovial test tubes (2 mL) were used as the center wells containing 0.5 mL 1% zinc acetate as trapping solution and filter paper (2 cm × 2.5 cm) to increase air:liquid contacting surface. The Erlenmeyer flasks containing the reaction mixture and center wells were flushed with N_2_ gas before being sealed with a double layer of parafilm. The reaction was initiated by transferring the flasks from ice to a 37°C shaking water bath. After incubating at 37°C for 90 min, 0.5 mL of 50% trichloroacetic acid was added to terminate the reaction. The flasks were incubated at 37°C for another 60 min to ensure a complete trapping of released H_2_S gas from the mixture. Contents of the center wells were then transferred to test tubes, each containing 3.5 mL of double distilled water. Subsequently, 0.5 mL of 20 mM *N,N*-dimethylphenylendiamine sulfate in 7.2 N HCl was added immediately followed by addition of 0.5 mL 30 mM FeCl_3_ in 1.2 N HCl. The reaction mixture was incubated in the dark for 20 min at room temperature. The absorbance of the resulting solution was measured at 670 nm in a Multiskan Spectrum (Thermo Labsystems).

Measurement of plasma H_2_S concentration was performed as described in pervious publications [29]–[31]. Aliquots of plasma samples (100 µL) were mixed with 1% zinc acetate and 12% NaOH (12%) and incubated in the dark for 15 min at room temperature. Subsequently, double distilled water (pH 12.8), 20 mM *N,N*-dimethylphenylendiamine sulphate in 7.2 N HCl, and 30 mM FeCl_3_ in 1.2 N HCl were added to the reaction mixture and incubated in the dark for 15 min at room temperature. The reactions were terminated by the addition of 10% trichloroacetic acid. Absorbance of the solution was measured at a wavelength of 670 nm in a Multiskan Spectrum (Thermo Labsystems). All samples were assayed in triplicate and H_2_S concentration was calculated against a calibration curve of NaHS.

### Measurement of Enzyme Activities

To determine FBPase activity, kidney homogenates were added to an assay mixture that contained 40 mM glycine buffer (pH 9.1), 1.0 mM EDTA, 2.0 mM MgCl_2_, 0.6 mM NADP^+^, and 1.2 U/mL of both glucose-6-phosphate dehydrogenase and phosphoglucose isomerase. The reaction mixture was equilibrated for 10 min at 37°C and initiated by the addition of 70 µM fructose-1,6-bisphosphate (F-1,6-P) and the increase in absorbance was measured at 340 nm in a Multiskan Spectrum (Thermo Labsystems), as described [32].

To determine phosphofructokinase (PFK) activity, kidney homogenates were added to an assay mixture that contained 50 mM Tris buffer (pH 8.0), 1.0 mM EDTA, 5.0 mM MgCl_2_, 2.5 mM dithiothreitol, 0.2 mM NADH, 1.0 mM fructose-6-phosphate (F-6-P), 1.5 U/mL aldolase, and 1.0 U/mL of both triosephosphate isomerase and glycerophosphate dehydrogenase. The reaction was initiated by the addition of 0.5 mM ATP and the decrease in absorbance was measured at 340 nm in a Multiskan Spectrum (Thermo Labsystems), as described [33].

### RNA isolation and Real-time quantitative PCR

Total RNA was isolated using RNeasy Mini Kit (Qiagen sciences, MD, USA) and followed accordingly to the manufacturer's instructions. First strand cDNA was prepared from total RNA (1 µg) by reverse transcription using iScript™ cDNA Synthesis Kit (Bio-Rad Laboratories, USA). Real-time quantitative PCR was performed on the iCycler iQ Real-time PCR Detection System (Bio-Rad, Nazareth). The primers specifically designed for mice are listed in Table 1. The rat primers, PGC-1α, ERRα, FBPase-1 and -2, and β-actin were predesigned by Qiagen Inc. (ON, Canada). The PCR conditions were as follows: denaturation at 95°C for 3 min, followed by 40 cycles of denaturation at 95°C for 30 s, annealing at 55°C for 1 min, and extension at 72°C for 30 s as described [3], [27], [28]. Specificity of the amplification was determined by melting curve analysis.

### Triosephosphates, F-1,6-P, and F-6-P analyses

Kidney homogenates were acidified with the addition of 1 N PCA (0.1 mM EDTA) for 5 min on ice, then centrifuged for 5 min at 12,000 rpm at 4°C. The supernatant was neutralized with 2.5 M K_2_CO_3_ and left to vent for 5 min on ice and was later centrifuged for 2 min at 12,000 rpm at 4°C. The supernatant was used for the metabolite assays described below.

The assay mixture to determine renal triosephosphates (GA3P and DHAP) and F-1,6-P levels contained 0.4 M triethanolamine buffer (pH 7.6), 40 mM EDTA, 34 µM NADH, 0.095 U/mL aldolase, 2.0 U/mL triosephosphate isomerase, and 0.3 U/mL glycerophosphate dehydrogenase. The reaction was initiated by the addition of the appropriate enzyme sequence and the decrease in fluorescence was measured at 355/440 nm *via* Fluoroskan Ascent (Thermo Labsystems) at as described [34].

The assay mixture to determine the renal level of F-6-P contained 0.4 M triethanolamine buffer (pH 7.6), 1 mM EDTA, 5 mM MgCl_2_, 0.6 mM NADP^+^, 1.0 U/mL glucose-6-phosphate dehydrogenase, and 1.7 U/mL phosphoglucose isomerase. The reaction was initiated by the addition of the appropriate enzyme sequence and the increase in fluorescence was measured at 355/440 nm *via* Fluoroskan Ascent (Thermo Labsystems) at as described [35].

### Chemicals and Data Analysis

All chemicals, primers, and enzymes used in this study were obtained from Sigma-Aldrich (Sigma Aldrich, ON, Canada). The data are expressed as mean ± s.e.m. All values presented as a percentage were normalized to the mean of age-matched CSE^+/+^ mice or untreated cells. Statistical analyses were performed using Student's *t* test, and when applicable, the one-way ANOVA followed by a *post hoc* analysis (Tukey's test). Statistical significance was considered at *P*<0.05.

## Results

### Reduced plasma glucose in 6-22 week-old CSE^-/-^ mice

**Figure 1:**
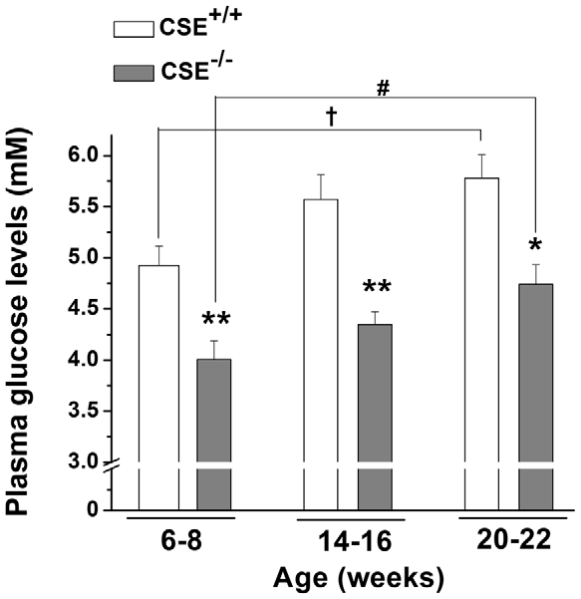
Plasma glucose levels in 6-22 week-old CSE^-/-^ mice. Plasma glucose levels were measured in 6-8 (*n* = 5–7), 14-16 (*n* = 4–5), and 20-22 (*n* = 6–7) week-old CSE^-/-^ and CSE^+/+^ mice after starving for 16 h. *P<0.05 and **P<0.01 vs. corresponding age-matched CSE^+/+^ mice; ^#^ *P*<0.05 vs. 6–8 week-old CSE^-/-^ mice; ^†^ *P*<0.05 vs. 6–8 week-old CSE^+/+^ mice**.**

The plasma glucose levels (mM) were significantly reduced by 18.64% in 6–8 (*P*<0.05), 21.94% in 14–16 (*P*<0.01) and 17.96% in 20-22 (*P*<0.05) week-old CSE^-/-^ mice in comparison to their age-matched WT mice under starvation conditions (16 h) (Fig. 1). Additionally, the plasma glucose levels were higher at 20-22 weeks of age compared to 6–8 weeks of age in both CSE^-/-^ and CSE^+/+^ mice (*P*<0.05).

### Elevated MG and reduced H_2_S levels in plasma and renal tissues in CSE^–/–^ mice

**Figure 2:**
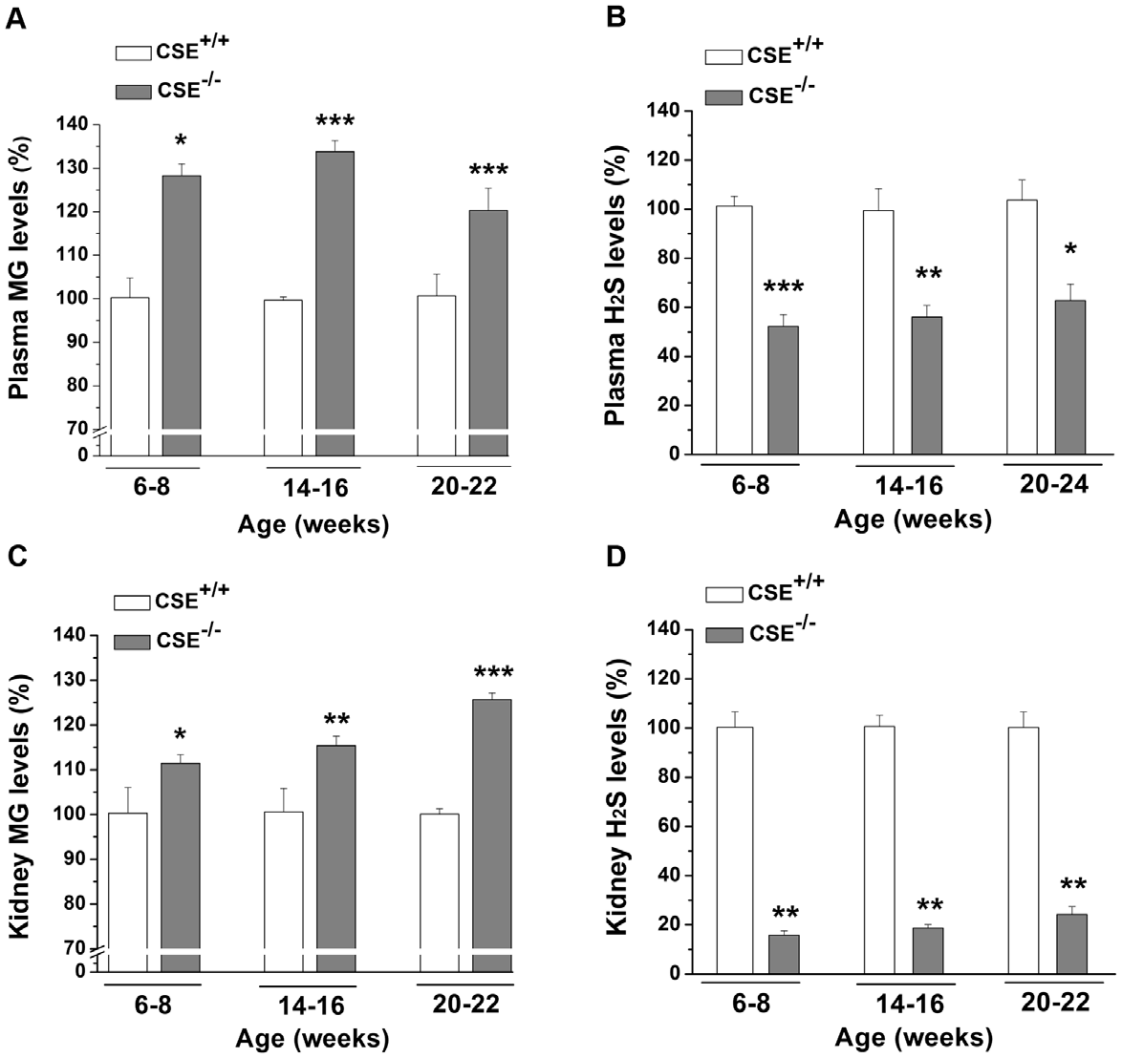
Methylglyoxal and H_2_S levels in plasma and renal tissues in CSE^–/–^ mice. **Panel A:** Methylglyoxal (MG) levels in the plasma where measured in 6-8 (*n* = 7), 14-16 (*n* = 4), and 20-22 (*n* = 7) week-old CSE^-/-^ and CSE^+/+^ mice. **Panel B:** H_2_S levels in plasma in 6-8 (*n* = 4-6), 14-16 (*n* = 4–6), and 20-24 (*n* = 3–5) week-old CSE^-/-^ and CSE^+/+^ mice. **Panel C:** MG levels in renal tissues of 6-8 (*n* = 5), 14-16 (*n* = 6), and 20-22 (*n* = 7) week-old mice. **Panel D:** Renal H_2_S levels in 6-8 (*n* = 3–5), 14-16 (*n* = 4), and 20–22 (*n* = 3-5) week-old mice. MG and H_2_S values from CSE^-/-^ mice are presented as a percentage of the mean of age-matched CSE^+/+^ mice. *P<0.05, **P<0.01, and ***P<0.001 vs. corresponding age groups of CSE^+/+^ mice.

Plasma MG levels (µM) were significantly increased in CSE^–/–^ mice by 28.28% in 6-8 (0.59±0.03, *P*<0.05), 33.80% in 14-16 (0.60±0.02, *P*<0.001), and 20.30% in 20-22 (1.85±0.05, *P*<0.001) week-old age groups with comparison to that from age-matched CSE^+/+^ groups (0.46±0.05, 0.42±0.01, and 1.54±0.04; respectively) (Fig. 2A). In addition, plasma H_2_S levels (µM) were significantly decreased by 47.76% in 6-8 (40.77±4.78, *P*<0.001), 43.40% in 14-16 (62.40±4.79, *P*<0.01), and 40.94% in 20-24 (82.81±6.69, *P*<0.05) week-old CSE^-/-^ mice compared to age-matched CSE^+/+^ mice (75.79±3.57, 111.41±8.98, and 131.96±16.60; respectively) (Fig. 2B). Kidney MG levels (nmoL/mg) were elevated by 11.41% in 6-8 (0.78±0.01, *P*<0.05), 15.40% in 14-16 (0.76±0.02, *P*<0.01), and 25.67% in 20-22 (0.68±0.01, *P*<0.001) week-old CSE^–/–^ mice compared to that from age-matched CSE^+/+^ mice (0.70±0.02, 0.66±0.03, and 0.54±0.01; respectively) (Fig. 2C). Negatively correlated to renal MG levels, kidney H_2_S production rate (nmoL/g/min) was significantly reduced by 84.47% in 6-8 (2.76±0.29, *P*<0.01), 85.61% in 14–16 (2.88±0.25, *P*<0.01), and 75.95% in 20-22 (3.23±0.46, *P*<0.01) week-old CSE^-/-^ mice compared to age-matched CSE^+/+^ mice (15.98±1.16, 14.22±1.74, and 13.33±1.41; respectively) (Fig. 2D).

### Impairment of FBPase activity and mRNA expression in 6-22 week-old CSE^–/–^ mice

**Figure 3:**
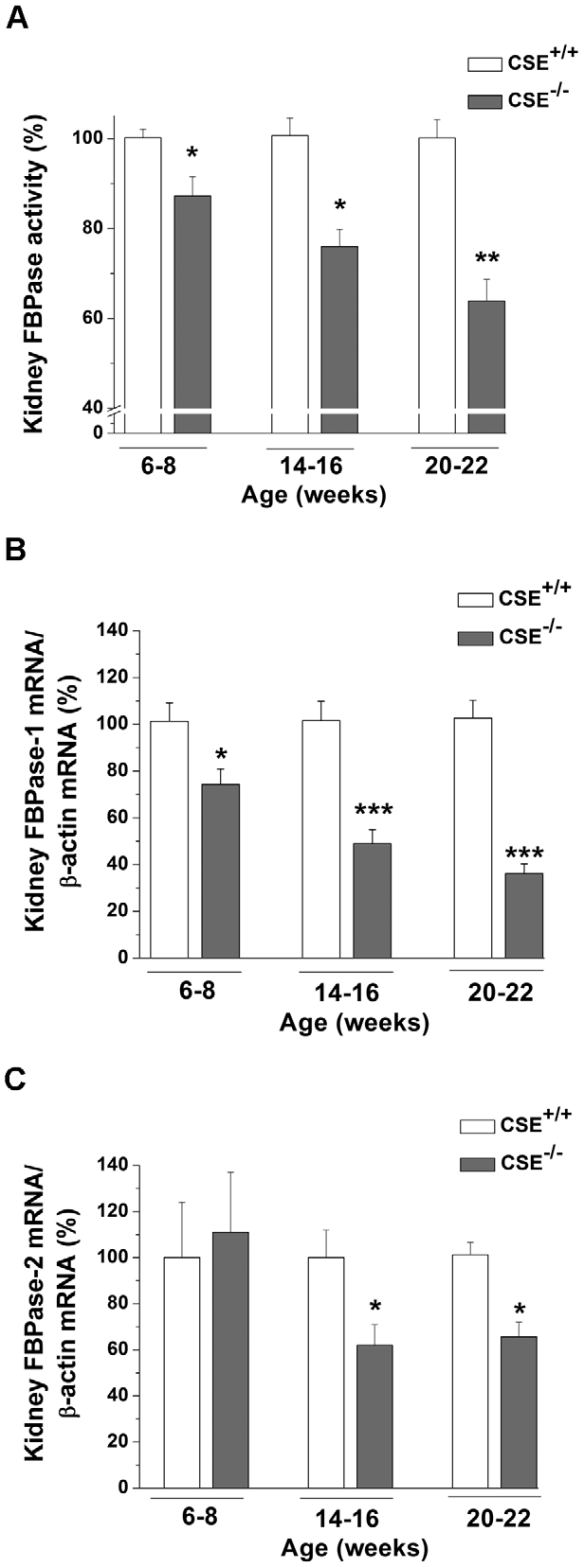
Total fructose-1,6-bisphosphatase activity and fructose-1,6-bisphosphatase-1 and -2 mRNA levels in renal tissues of CSE^–/–^ mice. **Panel A:** Total fructose-1,6-bisphosphatase (FBPase) activity in kidneys of 6-8 (*n* = 6), 14–16 (*n* = 6), and 20-22 (*n* = 4) week-old mice. **Panel B:** Real-time PCR results of fructose-1,6-bisphosphatase (FBPase)-1 levels in renal tissues of CSE^–/–^ mice ages 6-8 (*n* = 5), 14–16 (*n* = 7), and 20-22 (*n* = 5) weeks. **Panel C:** Real-time PCR results of FBPase-2 mRNA levels in kidneys of 6-8 (*n* = 4), 14–16 (*n* = 4), and 20-22 (*n* = 5) week-old mice. FBPase activity, FBPase-1 and -2 mRNA values in CSE^–/–^ mice are presented as a percentage of the mean of age-matched CSE^+/+^ mice. *P<0.05, **P<0.01, and ***P<0.001 vs. corresponding age groups of CSE^+/+^ mice.

Total kidney FBPase activity (nmoL NADPH/min/mg) was decreased by 12.74% in 6-8 (0.30±0.02, *P*<0.05), 23.98% in 14–16 (0.22±0.01, *P*<0.05), and 36.12% in 20-22 (0.16±0.01, *P*<0.01) week-old CSE^–/–^ mice compared to age-matched CSE^+/+^ groups (0.34±0.01, 0.29±0.03, and 0.24±0.01; respectively) (Fig. 3A). Similarly, we found that the FBPase-1 mRNA levels were decreased by 25.80±6.64% in 6-8 (*P*<0.05), 51.10±6.03% in 14-16 (*P*<0.001), and 63.90±4.20% in 20-22 (*P*<0.001) week-old CSE^–/–^ mice compared to that from age-matched CSE^+/+^ group (Fig. 3B). Although FBPase-1 was reported to be the dominant FBPase in the kidneys [36], [37], in one group of experiments we investigated whether FBPase-2 was expressed in CSE^+/+^ or altered in CSE^–/–^. We observed that FBPase-2 mRNA levels were expressed in both CSE^+/+^ and CSE^–/–^ mice. The mRNA levels of FBPase-2 were not changed in 6-8, but down-regulated by 38.05±9.12% in 14–16 (*P*<0.05) and 34.33±6.34% in 20-22 (*P*<0.05) week-old CSE^–/–^ mice compared to the control mice (Fig. 3C).

### Altered F-6-P, F-1,6-P, DHAP and GA3P levels in 6-22 week-old CSE^–/–^ mice

**Figure 4:**
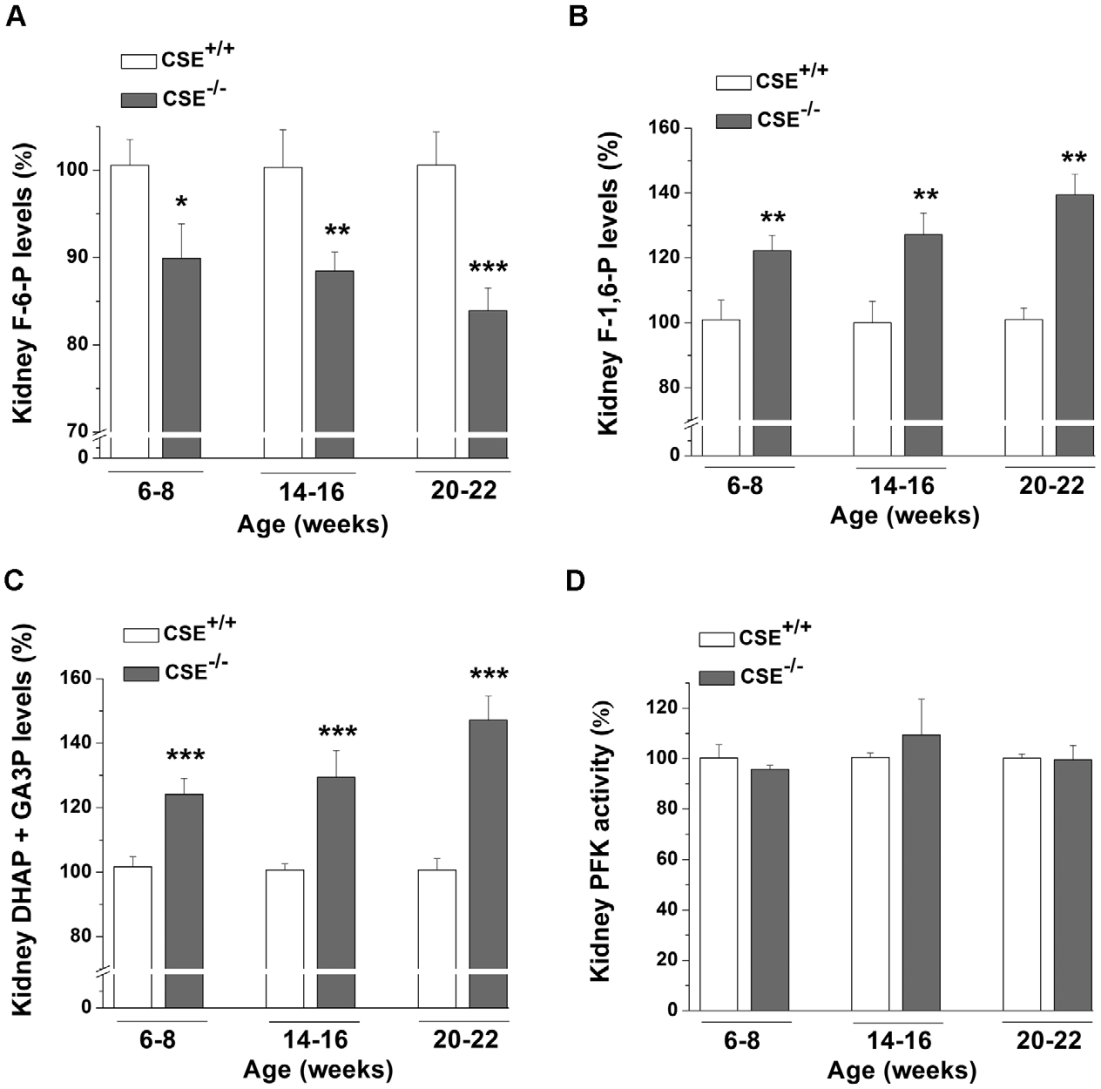
Fructose-6-phosphate, fructose-1,6-bisphosphate, and dihydroxyacetone phosphate and glyceraldehyde 3-phosphate levels in renal tissues of CSE^–/–^ mice. **Panel A:** Fructose-6-phosphate (F-6-P) levels were measured in kidneys of 6-8 (*n* = 6), 14–16 (*n* = 6), and 20–22 (*n* = 6-8) week-old mice. **Panel B:** Fructose-1,6-bisphosphate (F-1,6-P) levels in kidneys were analyzed in 6-22 (*n* = 5) week-old mice. **Panel C:** The triosephosphates, dihydroxyacetone phosphate (DHAP) and glyceraldehyde 3-phosphate (GA3P), were measured in kidneys of 6-22 (*n* = 5) week-old mice. **Panel D:** Renal phosphofructokinase (PFK) activity was measured in 6-8 (*n* = 4), 14–16 (*n* = 4–5), 20-22 (*n* = 4-5) week-old mice. The values from CSE^-/-^ are presented as a percentage of the mean of age-matched CSE^+/+^ mice. *P<0.05, **P<0.01, and ***P<0.001 vs. corresponding age groups of CSE^+/+^ mice.

The levels of F-6-P (nmoL NADPH/min/mg), the product of FBPase during gluconeogenesis, was decreased by 10.09% in 6-8 (1.51±0.05, *P*<0.05), 11.56% in 14–16 (1.49±0.03, *P*<0.01), and 16.07% in 20-22 (1.14±0.03, *P*<0.001) week-old CSE^–/–^ mice in comparison with that from age-matched CSE^+/+^ groups (1.68±0.04, 1.69±0.06, and 1.36±0.04; respectively) (Fig. 4A). In contrast, the levels of F-1,6-P (nmoL NADPH/10 min/mg), the substrate of FBPase, was increased by 22.21% in 6-8 (1.60±0.06, *P*<0.01), 27.14% in 14-16 (1.47±0.08, *P*<0.01), and 39.44% in 20-22 (1.50±0.11, *P*<0.01) week-old CSE^-/-^ mice compared to that from age-matched CSE^+/+^ group (1.31±0.06, 1.16±0.08, and 1.07±0.05; respectively) (Fig. 4B). Likewise, the renal DHAP and GA3P levels (nmoL NADH/10 min/mg), were also increased in CSE^–/–^ mice by 24.10% in 6-8 (1.44±0.06, *P*<0.001), 29.38% in 14-16 (1.55±0.09, *P*<0.001), and 47.14% in 20-22 (1.55±0.08, *P*<0.001) weeks old in comparison with that from age-matched CSE^+/+^ group (1.16±0.04, 1.20±0.04, and 1.05±0.04; respectively) (Fig. 4C). There was no change in the renal PFK activity, a glycolytic enzyme that coverts F-6-P to F-1,6-P, in both the CSE^–/–^ and CSE^+/+^ group at different age groups (Fig. 4D).

### Decreased mRNA expression of PGC-1α, PEPCK, and ERRα in 6-22 week-old CSE^–/–^ mice

**Figure 5:**
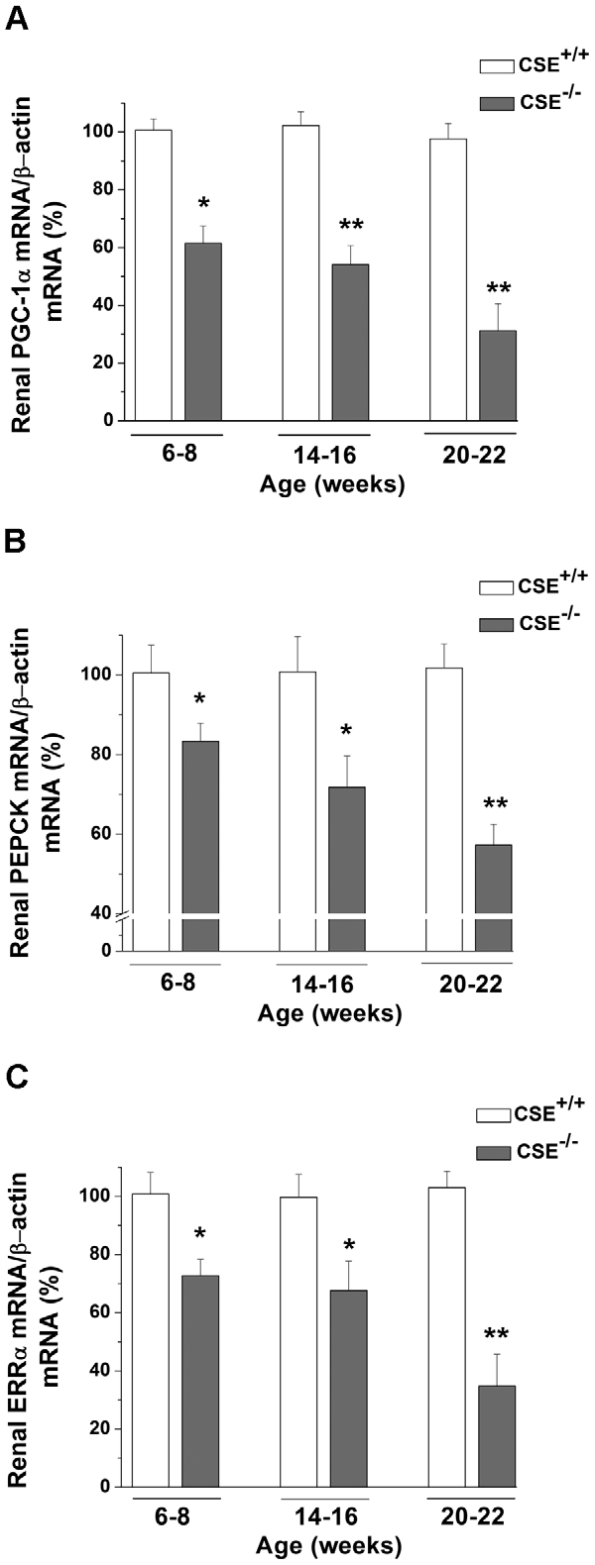
mRNA levels of peroxisome proliferator-activated receptor-γ coactivator-1α, phosphoenolpyruvate carboxykinase, and estrogen-related receptor-α in renal tissues of CSE^–/–^ mice. **Panel A:** Peroxisome proliferator-activated receptor-γ coactivator (PGC)-1α mRNA levels in kidneys of CSE^–/–^ mice in 6-8 (*n* = 5), 14–16 (*n* = 6), and 20-22 (*n* = 5) week-old mice. **Panel B:** Phosphoenolpyruvate carboxykinase (PEPCK) mRNA in 6-8 (*n* = 5), 14-16 (*n* = 6), and 20-22 (*n* = 5) week-old mice. **Panel C:** Estrogen-related receptor-α (ERRα) mRNA in 6-8 (*n* = 5), 14-16 (*n* = 6), and 20-22 (*n* = 5) week-old mice. The mRNA values from CSE^–/–^ mice are presented as a percentage of the mean of age-matched CSE^+/+^ mice. *P<0.05 and **P<0.01 vs. corresponding age groups of CSE^+/+^ mice.

Since PGC-1α was reported to be a major contributor to gluconeogenesis *via* the up-regulation of FBPase and PEPCK [21], the gene expression level of PGC-1α was investigated in these CSE^–/–^ mice. Interestingly, as shown in Fig. 5A, the mRNA levels of renal PGC-1α were lowered by 38.50±5.99% in 6-8 (*P*<0.05), 45.90±6.55% in 14-16 (*P*<0.01), and 68.78±9.35% in 20-22 (*P*<0.01) week-old age groups of CSE^–/–^ mice with comparison to age-matched CSE^+/+^ mice. Likewise, the renal mRNA levels of one of the down-stream targets of PGC-1α, PEPCK, were also decreased by 16.60±4.52% in 6-8 (*P*<0.05), 28.20±7.84% in 14-16 (*P*<0.05), and 42.70±5.18% in 20-22 (*P*<0.01) week-old CSE^–/–^ mice in comparison to that from age-matched CSE^+/+^ mice (Fig. 5B). Moreover, PGC-1α was also suggested to be a strong coactivator of ERRα [23], thus we sought to determine whether there was a corresponding fall in ERRα mRNA levels. Indeed, we observed lower mRNA levels of ERRα in the CSE^–/–^ mice by 27.70±5.65% in 6-8 (*P*<0.05), 32.40±10.10% in 14-16 (*P*<0.05), and 65.20±10.93% in 20-22 (*P*<0.01) weeks old compared to that from age-matched CSE^+/+^ groups (Fig. 5C).

### Down-regulation of FBPase-1 and -2 mRNA levels in aorta of CSE^-/-^ mice

**Figure 6:**
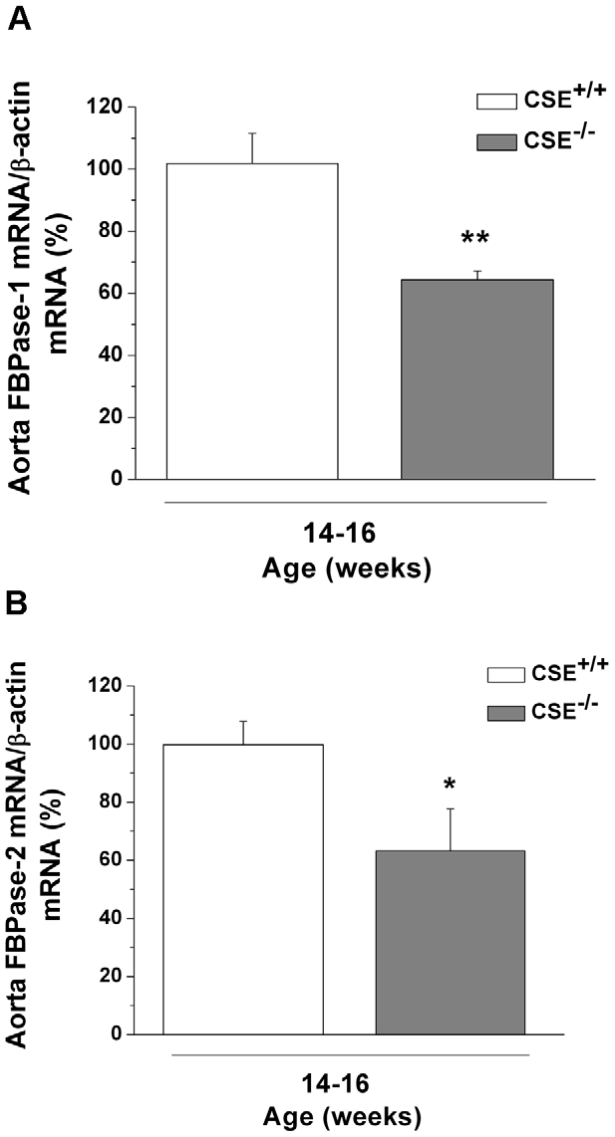
mRNA levels of FBPase-1 and -2 in the aorta of 14-16 week-old CSE^-/-^ mice. FBPase-1 **(Panel A)** and -2 **(Panel B)** mRNA expression levels were measured in aortic extracts from 14-16 week-old CSE^–/–^ mice (*n* = 3–4). The values from CSE^-/-^ are presented as a percentage of the mean of age-matched CSE^+/+^ mice. *P<0.05 and **P<0.01 vs. 14-16 week-old CSE^+/+^ mice.

In correlation to the observed decreased in renal mRNA levels of FBPase-1 and -2, we also reported a significant reduction of mRNA levels of FBPase-1 by 36.91±2.88% (*P*<0.01) and of FBPase-2 by 41.38±14.16% (*P*<0.05) in aorta tissues from 14-16 week-old CSE^-/-^ with comparison to that from age-matched CSE^+/+^ mice (Figs. 6A and B, respectively).

### H_2_S-induced up-regulation of PGC-1α, FBPase-1 and -2, and ERRα mRNA levels in A-10 cells

**Figure 7:**
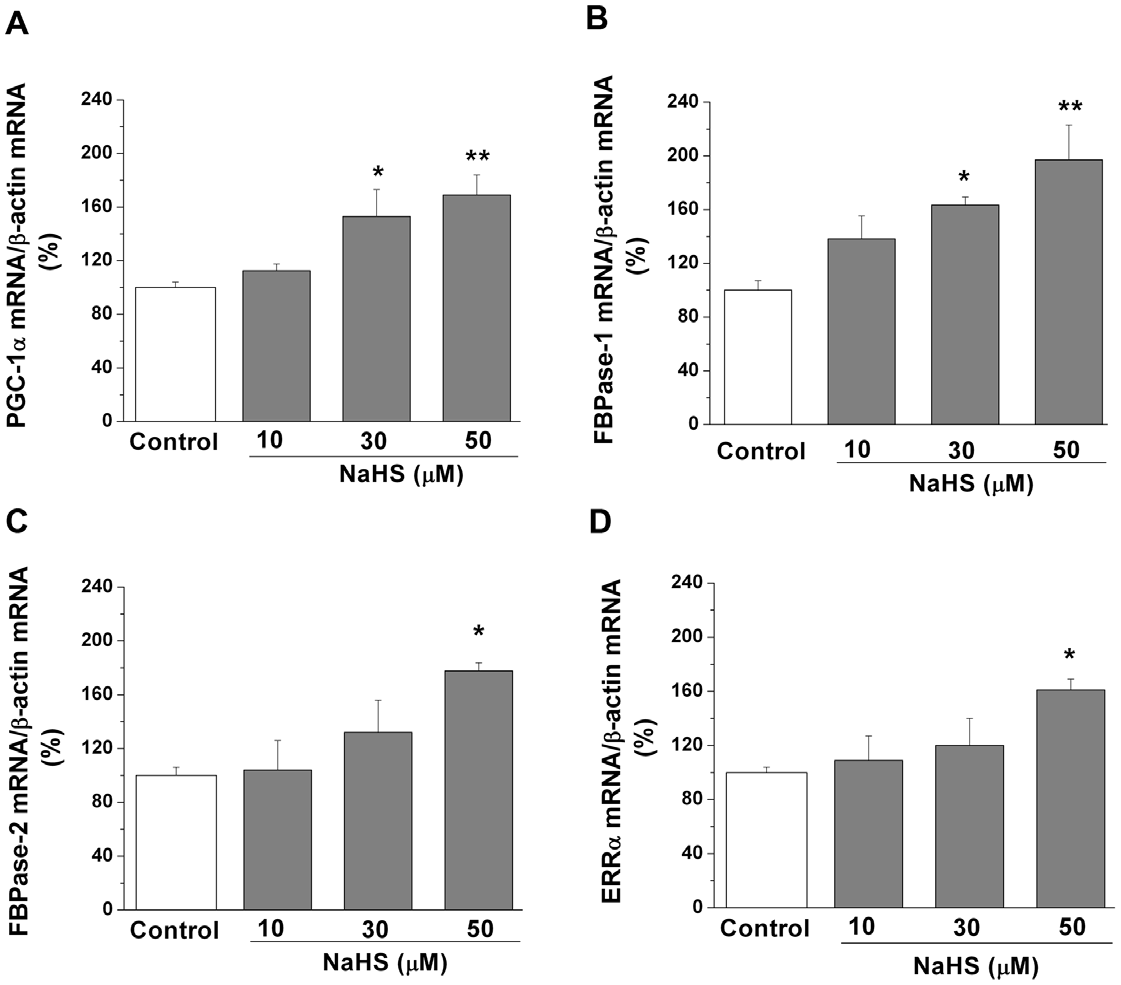
mRNA levels of proliferator-activated receptor-γ coactivator-1α, fructose-1,6-bisphosphatase-1 and -2, and estrogen-related receptor-α in NaHS-treated rat A-10 cells. Rat A-10 cells were treated with NaHS at different concentrations for 24 h to determine mRNA levels of proliferator-activated receptor-γ coactivator (PGC)-1α **(Panel A)**, fructose-1,6-bisphosphatase (FBPase)-1 **(Panel B)**, FBPase-2 **(Panel C)**, and estrogen-related receptor-α (ERRα) **(Panel D)**. *n* = 5 for each group in **Panels A**, **B**, **C**, and **D**. The mRNA values obtained from NaHS-treated A-10 cells are presented as a percentage of the mean of control cells. *P<0.05 and **P<0.01 vs. control group.

To determine if H_2_S directly up-regulated the gene expression of PGC-1α, ERRα, FBPase-1 and -2, rat VSMCs (A-10 cells) were subjected to different concentrations of NaHS, a H_2_S donor. We observed an increase in the mRNA levels of PGC-1α by 53.03±20.12% (*P*<0.05) and 69.21±15.18% (*P*<0.01) in the cells treated with 30 and 50 µM NaHS, respectively, in comparison with that of untreated cells (Fig. 7A). Correspondingly, we have also observed an up-regulation in the gene expression of FBPase-1 by 63.12±6.01% (*P*<0.05) in 30 µM NaHS- and 97.32±26.22% (*P*<0.01) in 50 µM NaHS-treated cells (Fig. 7B), along with FBPase-2 by 78.32±6.24% (*P*<0.05) and ERRα by 61.23±8.02% (*P*<0.05) in 50 µM NaHS-treated cells in comparison to the untreated cells (Figs. 7C and D, respectively).

## Discussion

The CSE^-/-^ mice are manifested with age-dependent hypertension [1] and increased proliferation of vascular smooth muscle cells [31]. In our previous studies, we also showed that H_2_S production was virtually eliminated in aortic tissues or smooth muscle cells from CSE^-/-^ mice [1], [31]. In the present study, we focused on the correlation of renal H_2_S metabolism and methylglyoxal formation. We found that H_2_S production from renal tissues of CSE^-/-^ mice was significantly suppressed in all three age groups (6–8, 14–16, and 20–22 weeks). This suppression of H_2_S production was accompanied by a parallel decrease in plasma glucose level, but sharply contrasted by an age-dependent increase in kidney and plasma MG levels in the CSE^-/-^ mice. Over the same observed age spectrum, CSE^-/-^ mice exhibited increased renal triosephosphates (DHAP and GA3P), the immediate precursors for MG formation [38]. These data indicate that reduced H_2_S production is likely linked to increased MG formation in the kidney of the CSE^-/-^ mice. Our study points to the underlying mechanism for this correlation to a down-regulated PGC-1α-FBPase signaling pathway in the kidney. Not only the transcriptional expression of PGC-1α and FBPase-1 and -2 was lower in CSE^-/-^ kidney, but also total FBPase activity and its metabolic product (F-6-P) were decreased. The importance of H_2_S level to the functionality of PGC-1α-FBPase signaling pathway is beyond the kidney. In aortic tissues from CSE^-/-^ mice, with low endogenous H_2_S production [31], lower mRNA levels of FBPase-1 and -2 were observed. On the other hand, the treatment of cultured rat aortic A-10 cells with an exogenous H_2_S donor, NaHS, significantly up-regulated the expression of PGC-1α, FBPase-1 and -2, and ERRα.

It has been extensively studied that abnormally high levels of H_2_S may be linked to T2DM and insulin-resistance [39]–[42], which could mainly be accredited to its ability to inhibit both insulin secretion from pancreatic β-cells [27], [43]–[45] and glucose-uptake into adipocytes [46]. Consequently, one would assume that this would affect the overall circulating glucose levels, where high H_2_S levels could lead to high systemic glucose levels. On the other hand, lower levels of H_2_S in the circulation and specific tissues are expected in favour of reducing plasma glucose levels and postponing the development of diabetes. Indeed, we have observed significantly lower plasma glucose levels (Fig. 1), as well as plasma H_2_S levels (Fig. 2B) in all three age groups of the CSE^-/-^ mice. Moreover, our recent study has reported that CSE^-/-^ mice that received streptozotocin injections exhibited a delayed onset of diabetic status (hyperglycaemia, hypoinsulinemia, and glucose intolerance), in comparison with wild type mice [28]. Thus, these findings further support the involvement of the CSE/H_2_S system in glucose regulation.

This is the first study to show that MG levels are elevated under reduced gluconeogenic conditions in plasma and renal tissues of the CSE knockout mice (Figs. 2A and C, respectively). The elevated renal MG levels, in turn, appear to be accounted for by the elevated renal levels of the MG precursors, DHAP and GA3P (Fig. 4C). This increased MG formation in kidneys of CSE knockout mice is important, as we have shown that elevated MG levels in kidneys of spontaneously hypertensive rats lead to increased advanced glycation endproducts formation and oxidative stress [13], [47]. The renal MG levels in the CSE^-/-^ mice were increased significantly in all three age groups (6–8, 14–16, and 20–22 weeks) when compared to age-matched CSE^+/+^ groups (Fig. 2C). Accompanied by the increased MG levels, renal H_2_S levels were significantly decreased in all three age groups (Fig. 2D); further supporting our hypothesis that MG is up-regulated in the presence of inadequate H_2_S.

FBPase is the rate-limiting enzyme in the gluconeogenic pathway and catalyzes the conversion of F-1,6-P to F-6-P [36]. There are two main isoforms of FBPase, FBPase-1 or liver FBPase, which is predominant in the liver and kidney [20], [36], [37], and FBPase-2 or muscle FBPase, which is predominant in skeletal muscle [48]. We observed lower activity of total FBPase in kidneys of 6-22 week-old CSE^–/–^ mice (Fig. 3A). The lower FBPase activity appears likely due to the down-regulation of the mRNA levels of FBPase-1 (Fig. 3B), and to a lesser extent, the down-regulation of FBPase-2 (Fig. 3C). As such, the decreased FBPase activity was accompanied by lower levels of its product, F-6-P, and higher levels of its substrate, F-1,6-P (Figs. 4A and B, respectively). To rule out possible interference from the glycolytic system, PFK activity, the enzyme responsible for the conversion of F-6-P to F-1,6-P, were measured. No changes in PFK activity were observed (Fig. 4D). These observations suggest that the down-regulation of FBPase was mainly responsible for the increased MG levels in renal tissues of CSE^-/-^ mice. A suppressed gluconeogenic system with increased MG formation in CSE^-/-^ mice is not only limited to the kidney. The mRNA levels of FBPase-1 and -2 were also down-regulated in aorta extracts from CSE^-/-^ mice (Figs. 6A and B, respectively).

The gene transcription of FBPase, along with PEPCK and ERRα, can be induced by PGC-1α [21]. PGC-1α is a critical regulator of genes related to energy metabolism [22]. As well, PGC-1α, along with ERRα, is abundant in human kidney, skeletal muscle, or tissues with high metabolic demand [49]. Interestingly, the endogenous gasotransmitters, NO [24] and CO [25], were shown to increase PGC-1α expression levels. In line with these previous observations, we show here for the first time that decreased H_2_S level in renal tissues (Fig. 2D) led to decreased PGC-1α gene expression (Fig. 5A), which most likely resulted in significantly lowered FBPase-1 and -2 mRNA levels and impaired FBPase activity in renal tissues of 6-22 week-old CSE^–/–^ mice (Fig. 3). In fact, both PEPCK and ERRα mRNA levels were also decreased in these renal tissues (Figs. 5B and C, respectively). Theoretically, decreased gene expression of PEPCK would reduce the formation of DHAP and GA3P, resulting in decreased MG formation. Our data of higher levels of DHAP, GA3P, and MG in renal tissues of CSE^-/-^ mice further indicate that it is the specific PGC-1α-FBPase pathway, rather than PGC-1α-PEPCK pathway, that contributes to the enhanced MG formation in these CSE deficient mice. In correlation to these findings in the mice, we have demonstrated that administration of 30 and 50 µM NaHS induced an increase in the mRNA expression levels of both PGC-1α and FBPase-1 in rat A-10 cells (Figs. 7A and B, respectively). Additionally, we have also observed a significant increase in FBPase-2 and ERRα mRNA levels in 50 µM NaHS-treated A-10 cells (Figs. 7C and D, respectively), further supporting the phenomenon of the involvement of H_2_S in the regulation of PGC-1α-FBPase pathway. Further molecular study is needed to determine how H_2_S regulates the gene transcription of PGC-1α.

**Figure 8:**
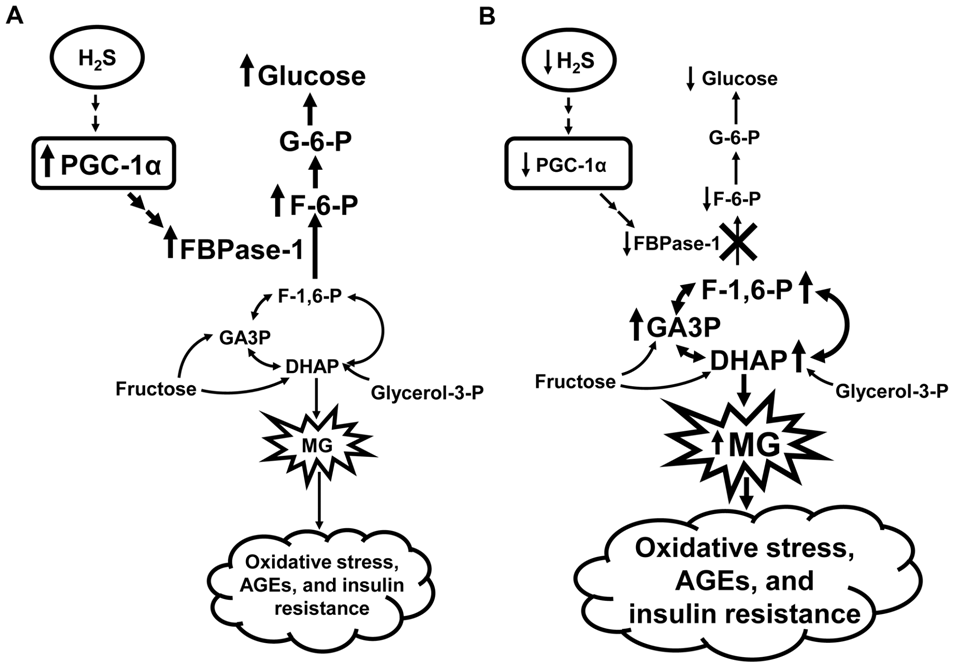
The regulatory effect of H_2_S on the direction of renal gluconeogenesis. **Panel A:** Gluconeogenesis with normal level of endogenous H_2_S. **Panel B:** Gluconeogenesis with lower level of endogenous H_2_S. AGEs: advanced glycation endproducts; DHAP: dihydroxyacetone phosphate; F-1,6-P: fructose-1,6-phosphate; F-6-P: fructose-6-phosphate; FBPase: fructose-1,6-bisphosphatase; G-6-P: glucose-6-phosphate; GA3P: glyceraldehyde 3-phosphate; H_2_S: hydrogen sulfide; MG: methylglyoxal; PGC-1α: peroxisome proliferator-activated receptor-γ coactivator-1α.

Our studies demonstrated that low levels of endogenous H_2_S in plasma and renal tissues from CSE^–/–^ mice (Figs. 2B and D, respectively) caused the down-regulation of PGC-1α, an important regulator of energy metabolism, along with some of its downstream targets, ERRα, PEPCK, and FBPase-1 and -2 in renal tissues (Fig. 8). Because the major unidirectional gluconeogenic enzymes, PEPCK and FBPase, are down-regulated, circulating glucose level is lowered. The decreased FBPase activity in the kidney of CSE^-/-^ mice encouraged the accumulation of MG precursors, DHAP and GA3P, which enhanced the formation of MG in both renal tissues and in the circulation. Henceforth, reduced endogenous H_2_S production attributes to the down-regulated PGC-1α-FBPase signaling pathway in mice lacking the CSE gene. As such, H_2_S redirects the gluconeogenesis process in the kidney from making glucose to producing MG, and sets up the balancing point between glucose and MG levels. The latter is likely exerting an important impact on renal functions.

**Table 1:** Real-time PCR primer sequences for gene targets in mice.

Gene name	Short name	Forward primer 5′-3′	Reverse primer 5′-3′
Peroxisome proliferator-activatedreceptor-γ coactivator-1α	PGC-1α	GGTACCCAAGGCAGCCACT	GTGTCCTCGGCTGAGCACT
Phosphoenolpyruvate carboxykinase	PEPCK	ATCTTTGGTGGCCGTAGACCT	GCCAGTGGGCCAGGTATTT
Estrogen-related receptor-α	ERRα	ATCTGCTGGTGGTTGAACCTG	AGAAGCCTGGGATGCTCTTG
Fructose-1,6-bisphosphatase-1	FBPase-1	CAGGGACGTGAAGATGAAGAAGAA	TTGTTGGCGGGGTATAAAAAGA
Fructose-1,6-bisphosphatase-2	FBPase-2	ACGTTATGGAAAAGGGGCGACAGG	GCTCCCCGAAATCCCATACAGGTT
β-actin	β-actin	CCCATCTACGAGGGCTAT	TGTCACGCACGATTTCC
